# Evaluation of a Semi-Automated Wound-Halving Algorithm for Split-Wound Design Studies: A Step towards Enhanced Wound-Healing Assessment

**DOI:** 10.3390/jcm13123599

**Published:** 2024-06-20

**Authors:** Paul Julius Georg, Meret Emily Schmid, Sofia Zahia, Sebastian Probst, Simone Cazzaniga, Robert Hunger, Simon Bossart

**Affiliations:** 1 Department of Dermatology, Inselspital University Hospital of Bern, University of Bern, 3010 Bern, Switzerlandsimon.bossart@insel.ch (S.B.); 2 Imito AG, 8047 Zurich, Switzerland; 3 Geneva School of Health Science, HES-SO University of Applied Sciences and Arts, Western Switzerland, 1206 Geneva, Switzerland; sebastian.probst@hesge.ch; 4 Care Directorate, University Hospital, 1206 Geneva, Switzerland; 5 Faculty of Medicine Nursing and Health Sciences, Monash University, Melbourne, VIC 3800, Australia; 6 College of Medicine Nursing and Health Sciences, University of Galway, H91 TK33 Galway, Ireland; 7 Centro Studi GISED, 24121 Bergamo, Italy

**Keywords:** chronic leg ulcers, wound assessment, split-wound design, automated measurement algorithm, inter–rater reliability, wound healing assessment, intra-rater reliability, evidence generation, cost savings

## Abstract

**Background**: Chronic leg ulcers present a global challenge in healthcare, necessitating precise wound measurement for effective treatment evaluation. This study is the first to validate the “split-wound design” approach for wound studies using objective measures. We further improved this relatively new approach and combined it with a semi-automated wound measurement algorithm. **Method**: The algorithm is capable of plotting an objective halving line that is calculated by splitting the bounding box of the wound surface along the longest side. To evaluate this algorithm, we compared the accuracy of the subjective wound halving of manual operators of different backgrounds with the algorithm-generated halving line and the ground truth, in two separate rounds. **Results**: The median absolute deviation (MAD) from the ground truth of the manual wound halving was 2% and 3% in the first and second round, respectively. On the other hand, the algorithm-generated halving line showed a significantly lower deviation from the ground truth (MAD = 0.3%, *p* < 0.001). **Conclusions**: The data suggest that this wound-halving algorithm is suitable and reliable for conducting wound studies. This innovative combination of a semi-automated algorithm paired with a unique study design offers several advantages, including reduced patient recruitment needs, accelerated study planning, and cost savings, thereby expediting evidence generation in the field of wound care. Our findings highlight a promising path forward for improving wound research and clinical practice.

## 1. Introduction

Chronic leg ulcers represent a significant challenge in healthcare due to their diverse etiologies and complex wound-healing dynamics [1,2,3,4]. Their prevalence is notable, and it is anticipated to rise in the future due to the aging population [5]. Effectively evaluating and comparing wound treatments, particularly in matched studies and randomized controlled trials, often demands a large number of patients for sufficient statistical power. This requirement complicates patient recruitment, lengthens study planning, and inflates costs.

Chronic leg ulcers are common, causing an enormous burden on patients, their families and the healthcare system, and they represent a major unmet medical need [1,2,6,7,8,9,10]. Novel methods to efficiently and objectively evaluate the therapeutic effects of wound treatments are urgently needed [11]. Wound surface area reduction is the central aim in the treatment of chronic skin wounds and a prime readout in the evaluation of the effectiveness of wound treatments [12,13,14,15,16]. However, intra- and inter-patient variability strongly influences the response of a given wound to a treatment. Variability is caused by the effects of age, gender, social status, ethnicity, comorbid medical conditions, the activities of daily living, healthy habits, and wound recurrence status on wound healing [1,17,18,19]. Consequently, the valid assessment of treatment effectiveness has remained a major challenge, requiring very large sample sizes to generate meaningful results [20].

Matching the need for low inter-patient variability, we hypothesize that it is possible to conduct wound studies using an in-wound split design. The current literature that uses a split-wound approach is mainly focusing on surgical wounds and scars [21,22]. Previous research of our group focused on halving the wound subjectively according to the clinical presentation to be able to treat both wound sides differently [6]. We further refined that method to create an objective algorithm that has a low variability. This algorithm should be able to split a wound into two equal areas and create a splitting line that can be transferred into real-world clinical applications.

To expedite wound healing, a wide range of topical wound products and cellular, acellular and matrix-like products (CAMPs) (e.g., Apligraf^®^, Epifix^®^, NuShield, Kerecis™ Omega3 Wound) are currently available. However, there remains a substantial unmet need in clinical practice for wound products that effectively and reliably support the healing of chronic leg ulcers based on evidence [11].

Another important factor of wound healing is the wound microbiome. The recent literature shows that wounds colonized with bacteria were more likely to become chronic compared to non-colonized wounds, and colonized wounds took significantly longer to heal compared to non-colonized wounds [23]. Analysis of 16S rRNA gene sequencing data revealed that distinct S. aureus strain types are associated with different wound outcomes. Certain strains were exclusively found in non-healing wounds, while other generalist strains were more broadly distributed across wounds [24].

A possible approach to compare skin replacement techniques involves treating one half of a chronic wound with surgical debridement and the other half with the experimental product or procedure, using surgical debridement as a control group. The analysis of the wound microbiome on treatment and control wound halves could also generate meaningful results. However, for meaningful comparison of wound halves, precise determination of the dividing line is essential.

The split-wound design introduces several key advantages in wound research. Traditional wound studies often require extensive patient recruitment efforts to find matched control subjects. This process can be time-consuming, costly, and challenging, particularly when dealing with a condition as variable as chronic leg ulcers. The split-wound design eliminates the need for finding comparable control subjects, as each patient serves as their own control. This significantly reduces the patient recruitment burden, making wound studies more feasible and faster to plan for researchers and clinicians.

Hereby, we aimed to assess the performance and reliability of a semi-automated wound-halving algorithm compared to manual halving for split-wound design studies.

## 2. Materials and Methods

An image software algorithm was designed to halve the wound along its longest side when placed within a rectangle. As most wounds are not perfectly round, there is typically a longer and a shorter side of the rectangle.

**Figure 1:**
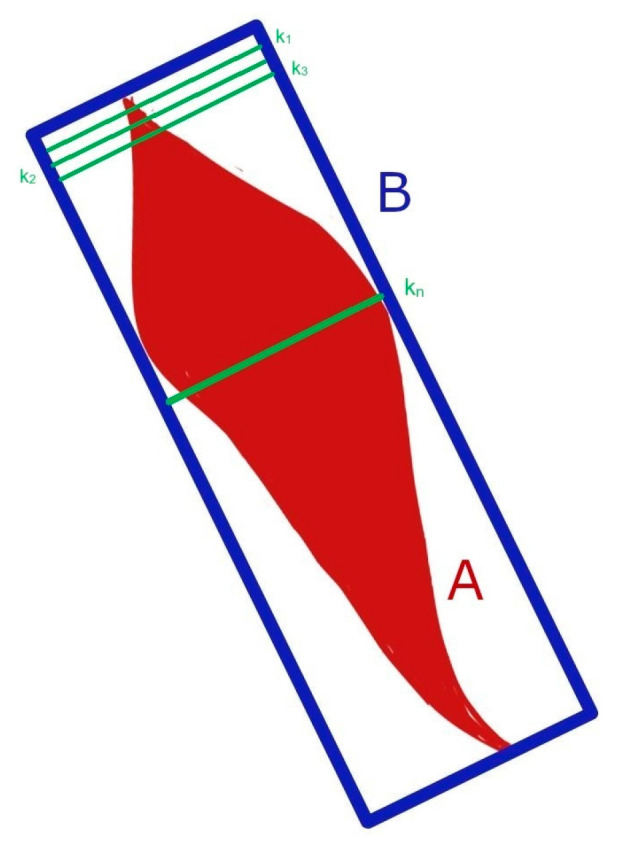
Approximation to 50% with 1-pixel wide steps.

**Figure 2:**
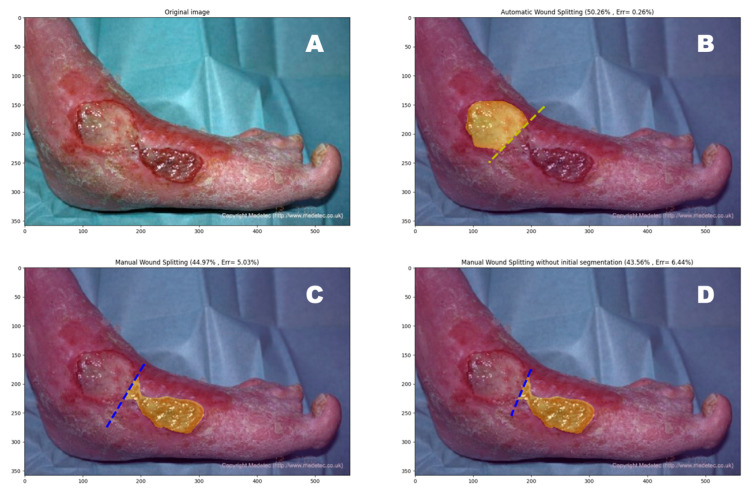
(**A**) An example of a leg ulcer (**B**) wound halving performed by the algorithm, (**C**) manual wound halving of round 1, and (**D**) manual wound halving of round 2.

The wound border was defined by an operator using an open-source image annotator [25]. Using the segmentation data, the algorithm identifies the longest length of the wound and constructs a bounding box encompassing the entire wound, aiming to minimize the area (Figure 1). It defines A as the wound and B as the enclosing rectangle. The algorithm calculates the total area of the large rectangle in pixels (B), the wound area (A), and the non-wound area in pixels (B-A). To find the midline, the algorithm iteratively creates small rectangles k_n_ with a width of n pixels and a length equal to the width of the wound area B, starting with n = 1. If the wound area within this small rectangle (A∩k_n_) does not approximate 50% of the wound area A, n is incremented by 1. This process continues until the area A∩k_n_ closely approximates 50% of the wound area A. The algorithm selects the rectangle k_n_ for which the wound area A∩k_n_ is closest to 50%. The length of this rectangle is then plotted on the wound image and serves as a template for wound halving (Figure 2).

### 2.1. Experimental Setup

We initially selected 20 images of chronic leg ulcer wounds of multiple origin, approximately 5–15 cm^2^ in size, available from two different open-source databases (6 images from DermNet (https://dermnetnz.org/topics/leg-ulcer-images (accessed on 1 August 2023)) and 14 images from Medetec Wound Database (https://www.medetec.co.uk/files/medetec-image-databases.html (accessed on 1 August 2023))). Surgical wounds and images not orthogonally framed were excluded.

Six assessors participated in the study, consisting of three wound experts (two dermatologists and one nurse) and three laypersons (two students and one statistician). The 20 images were provided to the assessors with a small handout explaining the use of the VGG Image Annotator (https://www.robots.ox.ac.uk/~vgg/software/via/via_demo.html, accessed on 1 September 2023, University of Oxford) to ensure consistent marking. After marking, the data were saved for subsequent analysis.

We conducted two analysis rounds with the same 20 previously selected images, with a four-week gap between the two rounds. The images were marked in a randomized order.

In the first round, assessors outlined the wound border on the ulcer images using the VGG Image Annotator. Subsequently, the assessors drew a midline through the wound using the same annotator. These subjective halving lines were compared with our algorithm-based halving line.

In the second round, assessors did not mark the wound edges using the VGG Image Annotator. Instead, they marked only the subjectively determined midline. These halving results were again compared with our algorithm-based wound halving.

To determine the wound edges for the algorithm, the assessors’ subjective wound outlining, drawn in the first round, was considered as 100% wound area, and the number of pixels in each half was calculated. The comparison was based on the absolute deviation from the ground truth calculation of the wound surface in pixels (Figure 1). The ground truth (line that is perfectly halving the wound) was defined as a wound surface percentage of 50%.

### 2.2. Statistical Analysis

Data were presented as medians with interquartile ranges (IQRs). Intra– and inter–rater reliability were assessed by using intraclass correlation coefficient (ICC) with two-way random, single measure, and absolute agreement [26].

ICC measures were presented with their 95% confidence intervals (CIs) and can be interpreted as follows: ≥0.80 excellent, 0.60–0.79 good, 0.40–0.59 moderate, 0.20–0.39 fair and <0.2 poor agreement [27].

The Wilcoxon signed-rank test was used to compare manual vs. automatic assessment. All tests were considered statistically significant at *p*-value < 0.05. Analyses were carried out with SPSS software v.26.0 (IBM Corp, Armonk, NY, USA).

## 3. Results

The median absolute deviation (MAD) from the ground truth of manual wound halving in the first round (with outlining), across all 20 images and all six assessors, was 2% (IQR = 1–4). In the second round (without outlining), the MAD across all images and assessors was 3% (IQR = 1–5) (Table 1).

Overall, we observed minimal deviation of the algorithm-generated halving line from the ground-truth-halving calculation (MAD = 0.3%, IQR = 0.2–0.5). The differences between the algorithm and the manual assessments were all statistically significant (*p* < 0.001). The inter–rater agreement, as assessed by ICC, was below 0.2 for all measurements.

The overall intra–rater agreement for before–after measurement was 0.3 (95% CI: 0.2, 0.5), varying from 0.2 to 0.6 among assessors (Table 2).

## 4. Discussion

In this comprehensive study, we introduced and evaluated a semi-automated wound measurement algorithm designed for split-wound design studies, which are a possible alternative for comparing wound treatments and interventions. Our analysis focused on assessing the algorithm’s performance and reliability in wound halving, comparing it to manual assessments, and considering its potential clinical applications.

Based on our analysis, we draw the following key conclusions.

Comparing manual and algorithm halving lines, the error of manual halving was higher (2–3% vs. 0.3%). In this setup, we did not compare different ways of wound border segmentation, which is an additionally relevant field impacting the calculation of the algorithm-based halving line [28]. The automation of wound border segmentation is a topic of ongoing research and different companies and institutions have developed artificial intelligence (AI) and traditional algorithms to target this topic [29]. Considering our wound border segmentation to be a reliable ground truth, we could show that there is a significant improvement in wound halving reliability looking at the absolute deviation from the ground truth. If we are focusing our analysis on the results of the ICC, we can see that the value for the semi-automated correlation (ICC = 0.2) does not show a better agreement than the wound halving in the first round with the aid of the graphically displayed wound border segmentation (ICC = 0.2). However, looking at the manual wound-halving agreement without guidance (second round), the correlation is even worse (ICC = 0.01). We can, therefore, conclude that the inter–rater agreement is highly impacted by wound segmentation and that manual wound halving without prior wound segmentation should not be adapted to conduct reliable wound studies.

These results demonstrate that our semi-automated wound-halving algorithm surpasses manual marking in terms of accuracy. The algorithm consistently produced halving lines that closely approximated the ground truth, with minimal deviation. In contrast, manual assessments, particularly when performed without the assistance of a graphically displayed wound border segmentation, exhibited significantly greater deviations from the ground truth. This finding underscores the value of this semi-automated algorithm in achieving reliable and consistent wound measurements, which are essential for clinical research and practice. To further improve accuracy, this method should be combined with an automated wound border segmentation.

A possible advantage is that this method enables direct measurements on both sides of the wound, facilitating the assessment of wound granulation and healing progress over time. The analysis of the wound microbiome in both wound sides could also be relevant information that can correlate to wound healing rates [23]. This direct comparison to the standard of care or control wound side enhances the ability to evaluate treatment efficacy and monitor potential adverse events, such as inflammation, eczema, or edema and other adverse reactions to the product. The comparison of the granulation tissue and scar quality after finished wound healing can provide valuable insights into the effects of interventions on wound healing dynamics.

However, it is essential to acknowledge certain limitations and considerations associated with this method. Variations in venous and lymphatic drainage between the proximal and distal wound sides can introduce potential confounding factors in split-wound studies. Researchers and clinicians should carefully account for these differences when designing and interpreting study outcomes. This potential confounder is minimized by the randomization of the wound intervention side and a degree of variation of the wound halving line plotting in almost round wounds.

Another potential limitation is wound–product diffusion. There is no guarantee that a wound product will affect only one side of the wound. Consequently, only products designed to limit diffusion should be used in split-wound studies to ensure the validity of treatment comparisons. To improve study quality, we therefore suggest the use of a wound divider in future study designs. This can consist of an individually formed stoma paste separator. Stoma paste has a thick and viscous consistency, making it easily moldable and suitable for creating a divider that can be adjusted to any wound. It does not interfere with wound healing and very rarely creates local irritation of skin or wounds. The analysis of the wound microbiome on either wound side should also be included in future study designs. Future improvements of this study approach should also consider 3D analysis of wounds to improve wound boarder segmentation and capture wound depth as a very relevant marker for wound healing assessment [30]. The analysis of the wound microbiome on either wound side should also be included in future study designs to address the confounding factor of wound colonialization [23].

In summary, this study provides evidence for the reliability and superiority of our semi-automated wound-halving algorithm over manual assessment, showing the importance of a reliable wound border segmentation. While certain limitations must be considered, the benefits of this method are clear, with the promise of advancing wound care and improving patient outcomes.

## 5. Conclusions

The validation of the semi-automated wound measurement algorithm, coupled with the split-wound design approach, herald a promising future for wound research and clinical practice. Chronic leg ulcers have long presented significant challenges, demanding precise wound assessment for effective treatment evaluation. The novel combination of the split-wound design and the semi-automated measurement algorithm addresses these challenges and offers numerous advantages.

The method also provides valuable insights into the effects of interventions on wound-healing dynamics, including granulation tissue and scar quality post-wound healing. By eliminating the need for matched controls, streamlining study planning, and offering cost savings, this approach paves the way for more efficient and impactful wound studies. While certain challenges exist, and the importance of reliable wound border segmentation has to be highlighted, the benefits are undeniable, promising advancements in wound care and improved patient outcomes. Future research should build upon these findings to further refine and expand the applications of automated split-wound measurement algorithms by automating wound border segmentation, which will soon be available in a number of proprietary software packages on the market. If it can be guaranteed that the diffusion of a wound product from the treatment into the control side can be limited, this method enables us to conduct objective and clinically relevant wound studies, minimizing bias.

## Figures and Tables

**Table 1:** Inter–rater agreement by the type of assessment.

Type	Absolute Value			Absolute Deviation	
	Median	IQR	ICC (95% CI)	Median	IQR
Algorithm	50	50–50	0.2	0.3	0.2–0.5
Manual 1st round	49	46–51	0.2	2	1–4
Manual 2nd round	50	47–53	0.01	3	1–5

CI: confidence interval, ICC: intraclass correlation coefficient, IQR: interquartile range.

**Table 2:** Before and after intra–rater agreement, overall and by individual examiner.

Round/		Absolute Value			Absolute Deviation	
Rater		Median	IQR	ICC 2 vs. 1	Median	IQR
				(95% CI)		
1st	1	49	46–55	-	4	2–6
	2	49	46–53		3	2–4
	3	48	46–50		3	1–4
	4	49	48–51		1	1–2
	5	48	46–50		2	1–4
	6	48	47–50		2	1–4
	All	49	46–51		2	1–4
2nd	1	52	48–55	0.5 (0.1, 0.7)	3	2–6
	2	50	48–54	−0.2 (−0.6, 0.3)	3	1–7
	3	50	46–54	0.2 (−0.2, 0.5)	4	2–5
	4	50	48–53	0.3 (−0.1, 0.6)	3	1–4
	5	50	47–53	0.6 (0.3, 0.8)	3	2–5
	6	50	47–52	0.6 (0.2, 0.8)	2	1–4
	All	50	47–53	0.3 (0.2, 0.5)	3	1–5

CI: confidence interval, ICC: intraclass correlation coefficient, IQR: interquartile range.

## Data Availability

The data presented in this study are available on request from the corresponding author.
